# Role of Lipoprotein(a) in Aortic Valve Calcification: Inflammatory and Oxidative Mechanisms Involved

**DOI:** 10.3390/ijms27156639

**Published:** 2026-07-25

**Authors:** Alberto Polo-Barranco, Carlos Rebolledo-Maldonado, Dairo Rodelo-Barrios, Juan Solano-Ropero, Valentina Rada-Obeso, Carlos Lavalle-Jiménez, Valeria Blanchar-Martínez, Carlos Beltran-Sánchez, Augusto Maza-Arnedo, Thalia Herrera-Calvo, Isaias Hazbun-Caicedo, Muna Isaac-Escorcia, José Correa-Guerrero, Elber Osorio-Rodríguez

**Affiliations:** 1 Faculty of Medicine, Universidad Metropolitana de Barranquilla, Barranquilla 080002, Colombia; dralbertopolob@gmail.com (A.P.-B.); carlos.rebolledo@unisimon.edu.co (C.R.-M.); valentinarada2003@gmail.com (V.R.-O.); 2 Group Sanitas Crea, Centros Médicos Colsanitas, Department of Internal Medicine, Clínica Colsanitas S.A., Clínica Iberoamérica, Barranquilla 080002, Colombia; 3 Group Care Medicine, Department of Intensive Medicine, Clínica Colsanitas S.A., Clínica Iberoamérica, Barranquilla 080002, Colombia; 4 Department of Critical Medicine and Intensive Care, Faculty of Medicine, Simón Bolívar University, Barranquilla 080002, Colombia; dairorodelo1992@gmail.com (D.R.-B.); juan.solanor@unisimon.edu.co (J.S.-R.); 5 Critical and Internal Medicine Research Group (CRITIMED), Cartagena de Indias 130005, Colombia; lavallecarlos44@gmail.com (C.L.-J.); valeriablanchar@gmail.com (V.B.-M.); augusto671maza@hotmail.com (A.M.-A.); taherrerac@hotmail.com (T.H.-C.); isaias651@outlook.com (I.H.-C.); dra.munaisaace@gmail.com (M.I.-E.); josegabriel2101@gmail.com (J.C.-G.); 6 Grupo Investigación en Ciencias de la Salud de la Universidad del Atlántico (GICS-UA), Faculty of Basic Sciences, Universidad del Atlántico, Puerto Colombia 081001, Colombia; carlosbeltran@mail.uniatlantico.edu.co; 7 Grupo Investigación Interdiciplinaria en Seguridad Alimentaria y Nutricional (GRIINSAN), Faculty of Health Sciences, Universidad del Atlántico, Puerto Colombia 081001, Colombia; 8 Faculty of Medicine, Corporación Universitaria Remington, Medellin 050010, Colombia; 9 Faculty of Medicine, Universidad del Sinú, Cartagena de Indias 130001, Colombia; 10 Department of Critical Medicine and Intensive Care, University of Cartagena, Cartagena de Indias 130005, Colombia

**Keywords:** lipoprotein(a), calcified aortic valve disease, aortic stenosis, oxidized phospholipids, autotaxin, valvular inflammation, oxidative stress, cardiovascular calcification

## Abstract

Elevated plasma lipoprotein(a) [Lp(a)] levels represent a predominantly genetically determined risk factor for atherosclerotic cardiovascular disease and calcific aortic valve disease (CAVD). Mechanistically, Lp(a) transports oxidized phospholipids, lysophosphatidylcholine, and autotaxin, components capable of promoting inflammation, oxidative stress, and valvular fibrocalcific remodeling. This review synthesizes the molecular, cellular, and clinical evidence linking Lp(a) to CAVD progression. Retention of Lp(a) and other apolipoprotein B-containing lipoproteins in the valvular matrix promotes endothelial activation, recruitment of cells of the monocytic lineage, and the release of proinflammatory mediators. Oxidized phospholipids and the autotaxin–lysophosphatidic acid axis activate redox-dependent pathways and promote the transition of valvular interstitial cells toward myofibroblastic and/or osteogenic phenotypes. These processes converge in alterations in cholesterol metabolism, the release of procalcifying extracellular vesicles, and hydroxyapatite nucleation. Genetic and imaging evidence supports an association between elevated Lp(a), microcalcifying activity, and accelerated hemodynamic progression. Although anti-Lp(a) therapies substantially reduce plasma Lp(a) concentrations, their effect on valvular outcomes has not yet been demonstrated.

## 1. Introduction

Plasma lipoprotein(a) [Lp(a)] concentrations are predominantly determined by genetic factors and show wide variability across different populations worldwide [1]. It is estimated that 10–30% of the global population has Lp(a) levels ≥ 50 mg/dL, with particularly high values observed among individuals of African descent [2,3]. From a biological perspective, Lp(a) combines a low-density lipoprotein (LDL)-like particle with apolipoprotein(a) and a significant load of oxidized phospholipids (OxPLs), characteristics that contribute to its atherogenic, pro-inflammatory, and potentially procalcifying properties [4,5,6]. Furthermore, its concentrations tend to remain relatively stable throughout life and are poorly modifiable by conventional lifestyle interventions, reinforcing its relevance as a marker of inherited risk and as a specific therapeutic target [7].

Elevated Lp(a) levels are an independent risk factor for atherosclerotic cardiovascular disease (ASCVD), ischemic stroke, peripheral arterial disease, and calcific aortic stenosis [8,9,10]. In patients with established ASCVD, the prevalence of Lp(a) > 50 mg/dL approaches 30%, highlighting its contribution to residual risk beyond traditional lipid factors [2]. Therefore, several scientific societies recommend measuring Lp(a) at least once during adulthood, especially in individuals with premature ASCVD, familial hypercholesterolemia, or a family history of early cardiovascular disease [7,11]. Although the strongest evidence links elevated Lp(a) levels to aortic valve calcification and aortic stenosis, emerging data suggest that Lp(a) may also be associated with other calcific valvular phenotypes, particularly mitral annular calcification [12,13]. However, these associations appear less consistent and less mechanistically defined than those observed in calcific aortic valve disease (CAVD); therefore, the present review focuses on the aortic valve [13].

Beyond its role in atherosclerosis, epidemiological, genetic, and imaging evidence has established Lp(a) as a factor closely linked to calcified aortic valve disease. Variants of the LPA locus, such as rs10455872, are associated with elevated Lp(a) concentrations and a significantly increased risk of incident aortic stenosis, supporting a causal relationship between this lipoprotein and valvular calcification [14,15]. Clinical studies have linked elevated Lp(a) concentrations to greater aortic-valve calcification burden, incident CAVD, and a higher likelihood of aortic valve replacement; however, associations with the rate of progression of established calcification are not fully consistent [10,12,16,17].

Although identifying individuals with elevated Lp(a) levels can improve cardiovascular and valvular risk stratification, specifically validated interventions to prevent aortic calcification or modify its hemodynamic progression are still lacking [18]. This limitation underscores the need to more precisely define the mechanisms that link Lp(a) retention in valvular tissue with inflammation, oxidative stress, and osteogenic activation of valvular interstitial cells (VICs) [18,19]. In particular, the contributions of OxPL, the autotaxin-lysophosphatidic acid axis, and nuclear factor kappa B (NF-κB) dependent proinflammatory pathways remain subjects of ongoing research [19]. Therefore, this review synthesizes the available molecular and clinical evidence on the role of Lp(a) in calcified aortic valve disease, with an emphasis on the inflammatory and oxidative mechanisms involved in its fibrocalcifying progression.

## 2. Structural and Molecular Role of Lp(a)

**Figure 1:**
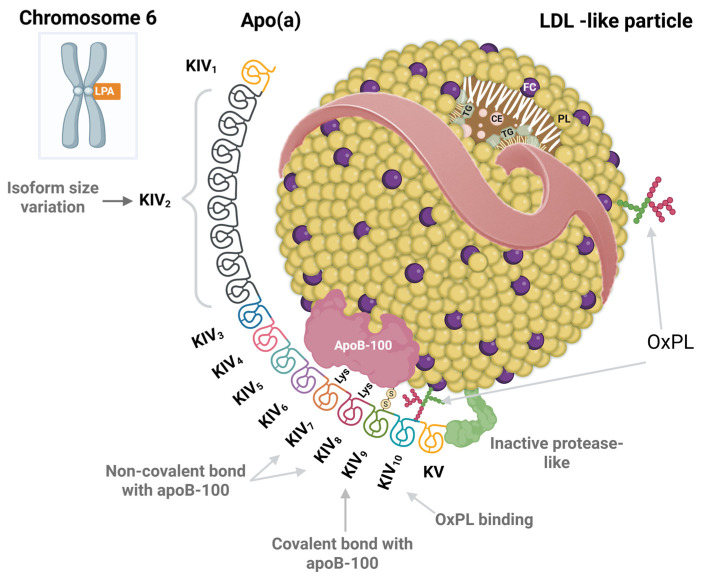
Structural scheme of Lp(a). Lp(a) consists of an LDL particle containing apolipoprotein B100 (apoB100) covalently linked via a disulfide bond (–S–S–) to apolipoprotein(a) [apo(a)]. Apo(a) is composed of multiple kringle domains—kringle IV (subtypes 1–10) and kringle V—and a catalytically inactive protease domain. The number of kringle IV type 2 (KIV-2) repeats varies widely among individuals and determines apo(a) size and, consequently, plasma Lp(a) concentrations. Abbreviations: LPA: apolipoprotein(a) gene; apoB100: apolipoprotein B100; apo(a): apolipoprotein(a); KIV: kringle IV; KIV-1: kringle IV type 1; KIV-2: kringle IV type 2; KIV-3: kringle IV type 3; KIV-4: kringle IV type 4; KIV-5: kringle IV type 5; KIV-6: kringle IV type 6; KIV-7: kringle IV type 7; KIV-8: kringle IV type 8; KIV-9: kringle IV type 9; KIV-10: kringle IV type 10; KV: kringle V; OxPL: Oxidized phospholipids. Adapted from: [26], Created in BioRender. Osorio, E. (2025) https://BioRender.com/wps73bu (accessed on 25 June 2026).

The *LPA* gene, located on chromosome 6q26-27, encodes apolipoprotein(a) [apo(a)], the distinctive component of Lp(a) [20]. Structurally, Lp(a) consists of an LDL-like particle containing apoB100, covalently linked to apo(a) via a disulfide bridge between cysteine 4326 of apoB100 and a cysteine located in the kringle IV type 9 domain of apo(a) [21,22]. The apo(a) gene shares marked structural homology with plasminogen, although it lacks kringle domains I, II, and III and primarily retains kringle domains IV and V [23]. Kringle domain IV is subdivided into 10 subtypes, from KIV-1 to KIV-10, among which KIV-2 exhibits extreme variability in the number of repeats, ranging from 1 to more than 40 copies between individuals [24]. This variability determines the size of the apo(a) isoforms such that smaller isoforms, with fewer KIV-2 repeats, are usually associated with higher plasma concentrations of Lp(a) [25]. See Figure 1.

The biological relevance of this structure is not limited to its similarity to plasminogen. Lp(a) is a preferential carrier of OxPL in human plasma. A single oxidized phospholipid may be covalently linked to apo(a), particularly within KIV-10, whereas most OxPL associated with Lp(a) are non-covalently bound and inserted into the lipid phase of the particle [27,28]. Although Lp(a) accounts for only a minor fraction of circulating apoB-containing particles, it carries the largest fraction of OxPL detected on apoB-containing lipoproteins, underscoring its disproportionate pro-inflammatory and procalcific potential [29]. Lp(a) can also transport autotaxin (ATX), an enzyme that converts lysophosphatidylcholine (LPC) into lysophosphatidic acid, a pathway implicated in inflammation, osteogenic differentiation of VICs, and aortic valve mineralization [19]. Furthermore, Lp(a) contains higher concentrations of LPC than other apoB-containing lipoproteins, which contributes to its pro-inflammatory effects on endothelial cells and macrophages [4,6]. These oxidative components provide a mechanistic basis for linking Lp(a) not only to atherosclerosis, but also to inflammation, osteogenesis, and increased valvular calcific activity [18,30].

Plasma Lp(a) concentrations are predominantly determined by genetic factors, with an estimated heritability between 70% and 90% [21]. Although variation in the number of KIV-2 repeats of the *LPA* gene accounts for a significant proportion of interindividual variability, other genetic modulators, such as apolipoprotein E (APOE) variants, may also influence plasma Lp(a) concentrations [31]. In general, the APOE ε2 allele has been associated with lower Lp(a) levels, whereas ε4-related associations appear more variable across populations; however, these effects are modest compared with the dominant influence of the *LPA* locus and apo(a) isoform size [32,33]. From a population perspective, individuals of African descent have Lp(a) concentrations approximately two to three times higher than those observed in European or Asian populations [24]. Differences according to sex and hormonal status have also been described: women may have higher concentrations than men at early ages, and Lp(a) levels increase after menopause, while postmenopausal hormone therapy has been associated with modest reductions in Lp(a) [34,35].

Unlike LDL-C, Lp(a) metabolism depends more on its rate of hepatic synthesis than on its peripheral catabolism [7,36]. Although several clearance pathways have been proposed, including hepatic uptake, LDL receptor (LDLR)-mediated internalization, lysosomal degradation, SR-BI involvement, and PlgRKT-mediated apo(a) recycling, their relative contribution to plasma concentrations appears limited compared with the genetic control of apo(a) production [31,37]. Thus, Lp(a) clearance appears to be conditioned by the particle’s three-dimensional architecture, including the apo(a) size isoform, determined by the number of KIV-2 repeats, and its potential influence on cell recognition, internalization, and the particle’s metabolic fate [38]. Taken together, these characteristics explain why Lp(a) behaves as a stable biomarker throughout life and as a predominantly inherited risk factor for atherosclerotic cardiovascular disease and calcified aortic stenosis.

Several mechanisms are shared between Lp(a)-mediated coronary atherosclerosis and CAVD, including apoB-particle retention, OxPL-driven inflammation, macrophage activation, and extracellular matrix remodeling [39,40]. However, the valvular process develops in a distinct biomechanical niche, where oscillatory shear stress, valvular endothelial dysfunction, matrix stiffness, and myofibroblastic/osteogenic differentiation of VICs shape fibrocalcific progression [41]. In contrast, coronary atherosclerosis is primarily organized around intimal plaque formation, lipid-core expansion, necrotic-core remodeling, and plaque rupture or erosion [40,42,43]. Consistent with this partial overlap, studies evaluating Lp(a) and coronary artery calcium (CAC) have shown that both elevated Lp(a) and CAC are independently associated with ASCVD risk, although their interaction appears limited and findings on CAC burden or progression remain heterogeneous [44,45].

## 3. Inflammatory Mechanisms

### 3.1. Inflammatory Activation of the Valvular Endothelium Mediated by Lp(a) and OxPL

In CAVD, the valvular endothelium acquires a pro-inflammatory phenotype characterized by increased permeability, endothelial dysfunction, and overexpression of adhesion molecules and chemokines, especially on the aortic side of the valve, which is exposed to oscillatory flow and non-uniform mechanical stress [46]. This hemodynamic microenvironment alters flow-sensitive transcriptional programs, including KLF2, KLF4, and Nrf2, reduces nitric oxide bioavailability, and promotes the activation of redox-dependent pathways associated with endothelial inflammation [47,48]. As a result, valvular endothelial cells (VECs) increase the expression of VCAM-1, ICAM-1, E-selectin, and MCP-1/CCL2, generating an adhesive surface that facilitates the recruitment, retention, and migration of inflammatory cells into the valvular interstitium [49]. This process represents an early stage of fibrocalcifying remodeling and promotes sustained interaction between hemodynamic, inflammatory, and lipid stimuli.

**Figure 2:**
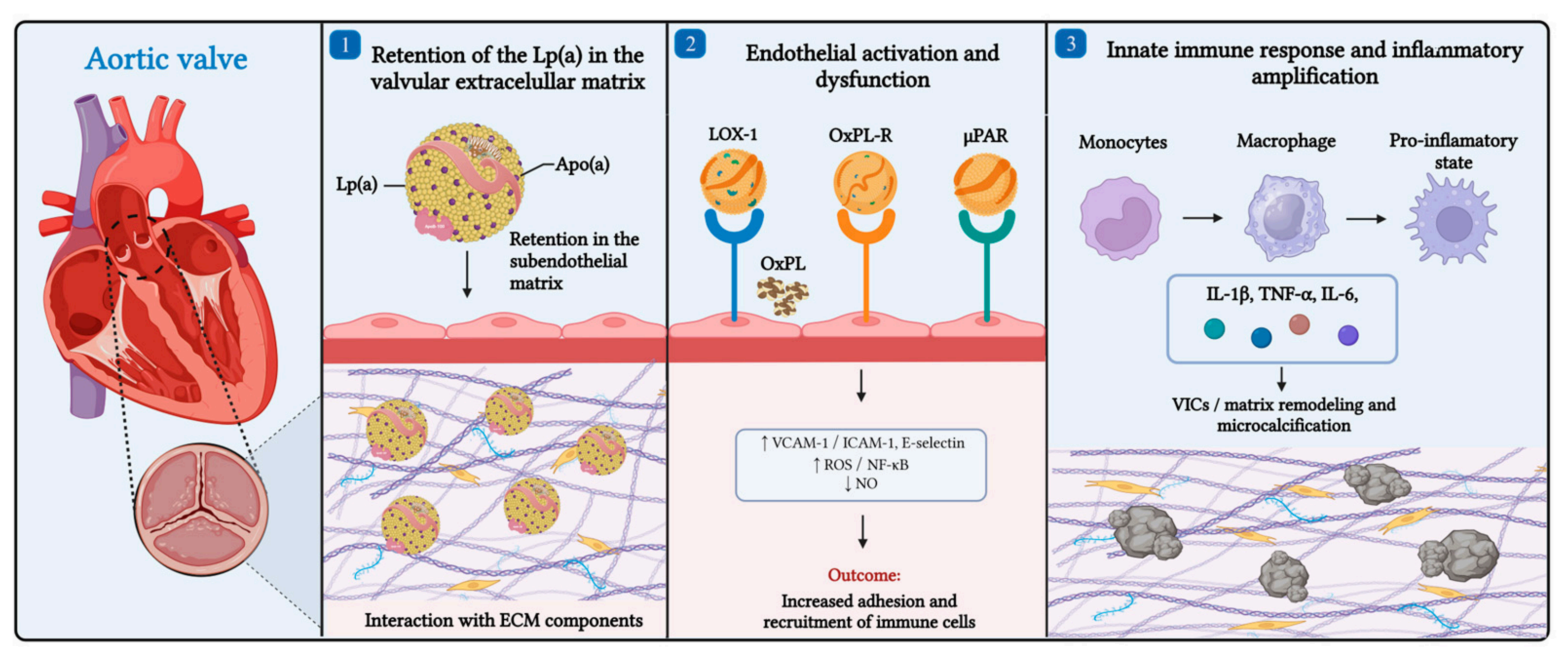
Inflammatory mechanisms linking lipoprotein(a) to aortic valve calcification. This schematic summarizes the main inflammatory pathways by which Lp(a) may contribute to CAVD. After retention within the valvular extracellular matrix, Lp(a) and its OxPL cargo promote endothelial activation, oxidative stress, and inflammatory signaling, favoring monocyte/macrophage recruitment and cytokine amplification. These events drive VICs activation, matrix remodeling, microcalcification, and progressive fibro-calcific remodeling of the aortic valve. Abbreviations: Apo(a): apolipoprotein(a); CAVD: calcific aortic valve disease; ECM: extracellular matrix; ICAM-1: intercellular adhesion molecule-1; IL-1β: interleukin-1 beta; IL-6: interleukin-6; LOX-1: lectin-like oxidized low-density lipoprotein receptor-1; Lp(a): lipoprotein(a); NF-κB: nuclear factor kappa B; NO: nitric oxide; OxPL: oxidized phospholipids; OxPL-R: oxidized phospholipid receptor; ROS: reactive oxygen species; TNF-α: tumor necrosis factor alpha; uPAR: urokinase-type plasminogen activator receptor; VCAM-1: vascular cell adhesion molecule-1; VIC(s): valvular interstitial cell(s). Created in BioRender. Osorio, E. (2026) https://BioRender.com/xpdarsl (accessed on 25 June 2026); figure preparation was completed in June 2026.

In this dysfunctional endothelium, the retention of apoB-rich lipoproteins, including Lp(a), is favored. The OxPL load of Lp(a) can amplify endothelial activation and increase the expression of mediators involved in leukocyte adhesion and chemotaxis [50,51]. The local accumulation of these particles can reinforce inflammatory communication among endothelial cells, VICs, and cells of the monocytic lineage, promoting the release of cytokines and chemokines such as TNF-α, IL-6, and MCP-1/CCL2 [52]. Thus, Lp(a) not only behaves as a lipoprotein retained in the valvular tissue, but also as a mediator that intensifies leukocyte recruitment and contributes to establishing a persistent inflammatory microenvironment [53]. See Figure 2.

### 3.2. Monocyte–Macrophage Infiltration and Osteogenic Transition of VICs

The endothelial phenotype activated in CAVD generates a chemotactic gradient dominated by MCP-1/CCL2, CX3CL1, and other chemokines, which favors the adhesion, migration, and infiltration of CD14+ monocytes into the valvular interstitium [54]. Once the endothelial barrier is crossed, these monocytes differentiate into macrophages with a dynamic functional spectrum that includes pro-inflammatory M1-like and reparative M2-like phenotypes [55]. However, the inflammatory microenvironment of CAVD is typically dominated by a pro-inflammatory profile, characterized by the production of IL-6, TNF-α, IL-1β, and ROS, which amplifies local inflammation and promotes pathological communication between immune cells and resident valve cells [56].

The uptake of Lp(a), oxLDL, and OxPL-enriched particles by scavenger receptors promotes the transformation of macrophages into foam cells, with intracellular accumulation of cholesterol esters and oxidized lipids [55]. These cells release matrix metalloproteinases, cathepsins, osteopontin, and additional chemotactic mediators, thereby promoting extracellular matrix degradation, disorganization of fibrillar architecture, and the formation of microenvironments prone to calcification [54]. In parallel, macrophage-derived proinflammatory cytokines act on VICs, inducing the expression of BMP2, RUNX2, and ALP and favoring their transition to an osteogenic phenotype, which constitutes a central axis of progressive valvular calcification [57]. See Figure 2.

### 3.3. Lp(a)–Autotaxin–Lysophosphatidic Acid Axis in Valvular Calcification

Lp(a) is characterized by transporting particularly high amounts of OxPL and LPC, which can serve as substrates for ATX, an ectoenzyme with lysophospholipase D activity involved in the generation of lysophosphatidic acid [19]. In CAVD, ATX has been identified on the valve surface and is associated with lipoproteins, where it catalyzes the conversion of LPC to lysophosphatidic acid, a bioactive lipid with pleiotropic effects on VECs, VICs, and macrophages [58]. This mediator activates G protein-coupled receptors, including LPAR1 and LPAR3, in the VICs, stimulating pathways such as RhoA/ROCK, PI3K/Akt, and MAPK, which promote cytoskeleton reorganization, increased myofibroblastic contractility, and the expression of inflammatory and osteogenic genes, including IL-6, IL-8, and BMP2 [59].

Furthermore, lysophosphatidic acid can enhance the synthesis of type I collagen and fibronectin, contributing to a stiffer, more fibrotic valve stroma and promoting osteoblastic differentiation of the VICs [60]. In the endothelial compartment, this axis can exacerbate VECs dysfunction by increasing permeability, reducing nitric oxide-dependent signaling, and enhancing the expression of adhesion molecules [19]. Thus, the Lp(a)–autotaxin–lysophosphatidic acid axis integrates lipid signaling, inflammation, fibrosis, and osteogenic remodeling, constituting a functional bridge between OxPL accumulation and the fibrocalcifying transition of the aortic valve [58]. See Figure 2.

### 3.4. Activation of NF-κB and Procalcifying Cytokines

OxPL and lysophosphatidic acid generated by the ATX-LPC pathway converge in the activation of redox-dependent inflammatory pathways, including NF-κB, in both VECs and VICs [51,53]. In VICs, NF-κB activation promotes an inflammatory program associated with increased expression of IL-6, IL-1β, and TNF-α, along with mediators involved in extracellular matrix remodeling and mineralization, including BMP2, RUNX2, and osteopontin [46,61]. IL-6, through classical and trans signaling of the IL-6/IL-6R/gp130 axis, can activate the JAK/STAT3 pathway and reinforce the inflammatory-osteogenic transition of VICs, contributing to the progression of the calcifying phenotype [53,62].

This NF-κB–IL-6–BMP2 circuit can act as a molecular amplifier that promotes the progressive stabilization of VICs to an osteoblastic phenotype, promotes the synthesis and remodeling of a type I collagen-rich matrix, and alters the balance between procalcifying signals and endogenous anticalcifying mechanisms, including matrix Gla protein [61]. Furthermore, persistent inflammation can modulate the epigenetic status of valve cells through changes in histone acetylation, DNA methylation, and the expression of microRNAs associated with inflammation and osteogenesis, contributing to the maintenance of a fibrocalcifying program even in the presence of fluctuating stimuli [46,63]. Taken together, this inflammatory network links OxLDL retention with osteogenic activation of valve cells and the structural progression of CAVD. See Figure 2.

## 4. Oxidative and Osteogenic Mechanisms

### 4.1. Pro-Inflammatory and Pro-Calcifying Effects of OxPL Associated with Lp(a)

Lp(a) concentrates a disproportionate fraction of OxPL compared to other apoB-rich lipoproteins, partly due to the presence of apo(a) and its kringle domains, which facilitate the association of these bioactive lipids with the particle [18]. This OxPL load gives Lp(a) a highly pro-inflammatory and pro-calcifying profile, capable of linking lipid retention with inflammatory and osteogenic activation of valvular tissue [64]. In the aortic valve, Lp(a) and other apoB-containing particles can be retained in the extracellular matrix through interactions with proteoglycans, collagen fibers, and other components of the valve matrix [18,62]. Once retained, OxPL associated with these particles can be recognized by scavenger receptors, including LOX-1, CD36, and SR-A, expressed on VECs, VICs, and macrophages [65].

**Figure 3:**
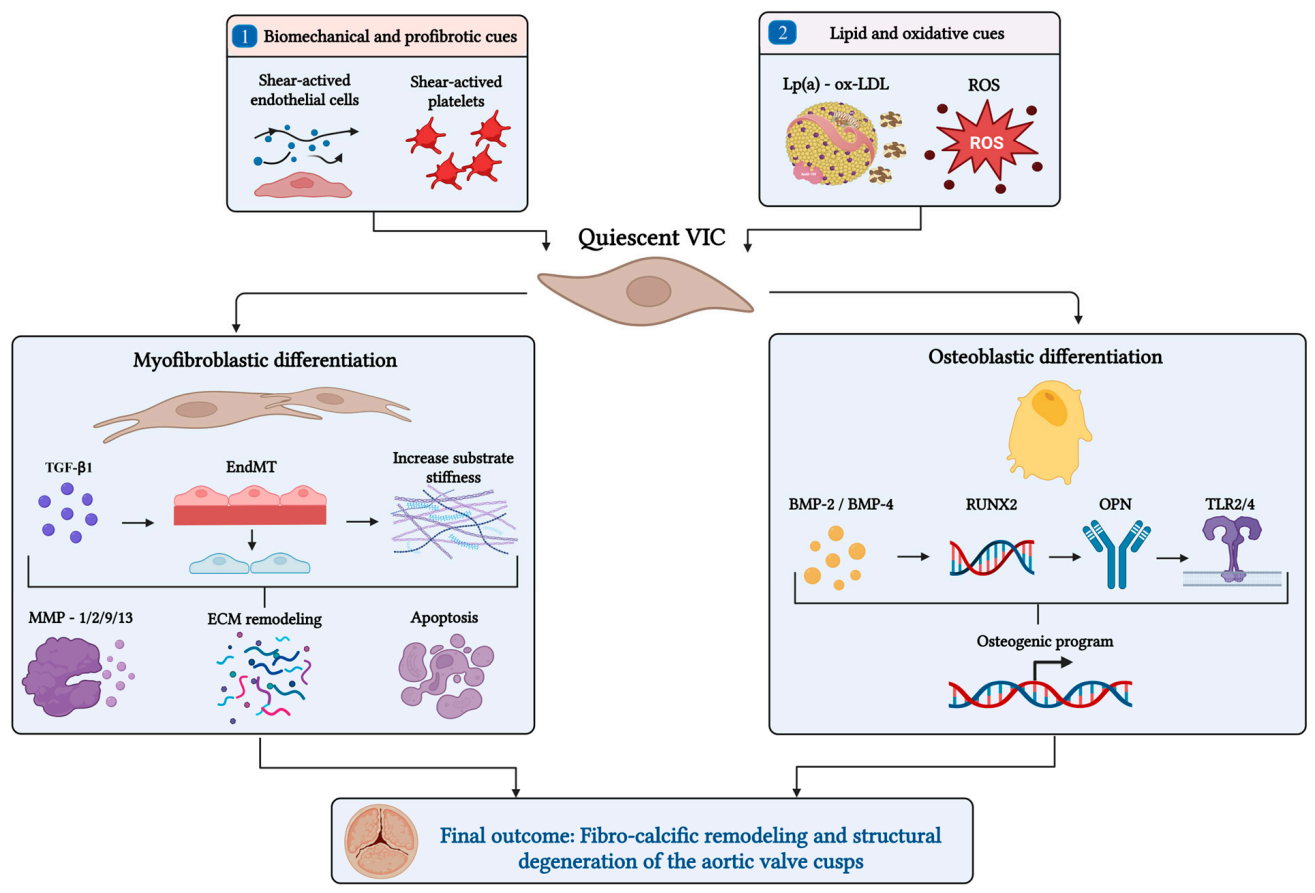
Phenotypic transition of valvular interstitial cells in calcific aortic valve disease. This schematic illustrates the transition of quiescent VICs toward either myofibroblastic or osteoblastic phenotypes in CAVD. Biomechanical, inflammatory, lipid, and oxidative cues promote profibrotic and osteogenic signaling, leading to matrix remodeling, apoptosis, osteogenic programming, and calcific nodule formation. Together, these processes converge on fibro-calcific remodeling of the aortic valve. Abbreviations: BMP: bone morphogenetic protein; CAVD: calcific aortic valve disease; ECM: extracellular matrix; EndMT: endothelial-to-mesenchymal transition; Lp(a): lipoprotein(a); MMPs: matrix metalloproteinases; OPN: osteopontin; ox-LDL: oxidized low-density lipoprotein; ROS: reactive oxygen species; RUNX2: runt-related transcription factor 2; TGF-β1: transforming growth factor beta 1; TLR2/4: Toll-like receptor 2/4; VIC(s): valvular interstitial cell(s). Created in BioRender. Osorio, E. (2026) https://BioRender.com/qwdd4ey (accessed on 25 June 2026); figure preparation was completed in June 2026.

Signaling induced by these OxLDLs increases ROS production, activates redox-dependent inflammatory pathways, and promotes the expression of oxidative enzymes, including NADPH oxidase and xanthine oxidase [66]. Furthermore, OxPLs can alter cell membrane organization, modify lipid microdomains, and modulate calcium-dependent signaling—mechanisms that affect cell viability, the inflammatory response, and the propensity for mineralization [67]. Thus, the oxidized fraction associated with Lp(a) acts simultaneously as an inflammatory, oxidative, and procalcifying stimulus in the valvular microenvironment [68]. Taken together, these processes favor the fibrocalcifying transition characteristic of CAVD and are associated with a greater burden of valvular calcification and with the progression of aortic stenosis [18]. See Figure 3.

### 4.2. Oxidative Stress and Profibrotic and Osteogenic Axes TGF-β1/BMP2/RUNX2

Valvular oxidative stress results from the convergence of mitochondrial ROS; activation of NADPH oxidases, particularly the Nox2 and Nox4 isoforms; and eNOS uncoupling in the context of chronic inflammation and lipid overload [62]. These ROS not only damage components of the extracellular matrix and DNA but also act as second messengers that modulate profibrotic and procalcifying pathways, including TGF-β1 and BMP2 [51]. TGF-β1 promotes myofibroblastic differentiation of VICs, with increased α-SMA, cell contractility, and collagen synthesis; however, its effect on mineralization may depend on the experimental model and the biological context [69]. In parallel, BMP2 participates in the osteogenic programming of the VICs and, together with RUNX2, promotes the expression of genes associated with valve mineralization, such as alkaline phosphatase (ALP), osteocalcin, and osterix [61].

The oxidative environment may also facilitate the activation of the Wnt/β-catenin and Notch pathways, which interact with the TGF-β/BMP and RUNX2 axes to amplify the osteogenic phenotype of the VICs [57]. In addition, oxLDL can disrupt cholesterol homeostasis in VICs and promote intracellular cholesterol accumulation within the valvular microenvironment [62]. Recent evidence indicates that oxLDL induces FOXS1 and alters the BSCL2/PPARγ/LXRα pathway, reducing the expression of cholesterol efflux transporters such as ABCA1 and ABCG1; this process promotes lipid transport dysfunction and inflammasome-mediated inflammation [70]. Thus, the oxLDL–FOXS1–BSCL2/PPARγ/LXRα–NLRP3 axis connects lipid dysfunction, innate inflammation, and progressive valvular calcification [70]. See Figure 3.

### 4.3. Transformation of Valvular Interstitial Cells Towards an Osteoblastic Phenotype

VICs constitute the central effector population in valvular calcification, due to their ability to modify the extracellular matrix and adopt phenotypes activated by inflammatory, mechanical, and lipid stimuli [53]. Under physiological conditions, these cells maintain a predominantly quiescent phenotype, with low proliferative activity and functions oriented towards maintaining valvular architecture and extracellular matrix homeostasis [71]. In CAVD, sustained exposure to Lp(a)/OxPL, oxLDL, lysophosphatidic acid, proinflammatory cytokines, TGF-β1, and BMP2 promotes a progressive transition of VICs toward activated phenotypes, integrating lipid, inflammatory, oxidative, and osteogenic signals [51,53,57]. Initially, these cells acquire myofibroblastic characteristics, with increased α-SMA, contractility, and fibrous matrix synthesis; Subsequently, they can progress to an osteoblastic phenotype, characterized by the expression of ALP, RUNX2, BMP2, osterix, and bone matrix proteins [61].

In parallel, endothelial-mesenchymal transition processes can contribute to the expansion of cells with mesenchymal characteristics, similar to VICs activated from VECs, thereby expanding the cellular reservoir susceptible to participating in osteogenic programs [46]. The combination of matrix stiffness, mechanical stress, proinflammatory signaling, and oxLDL-derived stimuli establishes a feedback loop that favors the persistent activation of VICs and the progression of valvular fibrocalcifying remodeling [72]. In addition, activated VICs can release procalcifying extracellular vesicles (EVs), cytokines, and matrix remodelers, contributing to mineral nucleation, perpetuation of local inflammation, and expansion of calcification into adjacent regions of the valve [73]. However, it is important to note that the osteoblastic pathway, although well characterized in experimental and in vitro models, may have a more limited histological representation in clinical CAVD [74,75]. True ossification foci have been reported in only a minority of surgically excised valves, indicating that osteogenic signaling contributes to disease progression even when mature bone formation is not present [76]. See Figure 3.

### 4.4. Calcification Mediated by Extracellular Vesicles and Hydroxyapatite Formation

Importantly, valvular calcification is not solely the consequence of extracellular matrix degradation or passive calcium deposition [39,62]. Rather, it is an active process involving lipid accumulation, inflammation, extracellular matrix remodeling, cell death, and the release of mineralization-competent EVs [39,77]. Early mineral nucleation may occur around apoptotic bodies, EVs, necrotic cell-derived debris, and acidic phospholipid-rich cellular remnants, which provide surfaces capable of binding calcium and phosphate [77,78,79]. These structures may act as initial platforms for hydroxyapatite nucleation and microcalcification growth, thereby linking cell death, lipid accumulation, and matrix remodeling to the early stages of fibrocalcific disease [79,80].

Valvular calcification does not arise solely from passive calcium precipitation, but is actively organized around EVs, released by VICs, activated VECs, and macrophages [81]. These EVs can act as precursors of microcalcifications in fibrocalcifying tissue and participate in valvular mineralization from early stages [77]. They include microvesicles originating from plasma membrane budding and exosomes derived from multivesicular bodies, whose contents can be enriched with tissue ALP, annexins, phosphatidylserine, phosphate regulators, and matrix proteins associated with calcification [82].

The exposure of phosphatidylserine and the presence of tissue ALP on the surface of these vesicles promote the hydrolysis of pyrophosphate, a physiological inhibitor of mineralization, and facilitate the nucleation of hydroxyapatite crystals in the extracellular matrix [83]. VEs are preferentially deposited in regions with disorganized matrix, enriched in type I collagen and fragmented elastin, where they act as nuclei of mineralization capable of expanding and coalescing into macroscopic calcified plaques [77,84]. Furthermore, the protein, lipid, and transcriptomic profile of vesicles retained in calcified valves reflects the inflammatory, redox, and procalcifying state of the tissue of origin, supporting their role as active mediators of fibrocalcifying remodeling and as potential translational biomarkers of CAVD [85]. See Figure 3.

### 4.5. Interactions with OxLDLs and Small, Dense LDL

Although Lp(a) is a central player in CAVD, it does not act in isolation [7]. The valve is exposed to a complex lipid microenvironment, with retention of apoB-containing lipoproteins, including LDL, remnant particles, and modified or oxidized forms, which contribute to local inflammatory and fibrocalcifying remodeling [53]. Small, dense LDL particles are more susceptible to retention in proteoglycan-rich matrices and oxidative modification [86]. Following oxidation, the phospholipids of these particles can generate LPC and other bioactive lipids; Lysophosphatidylcholine can serve as a substrate for ATX, promoting the formation of lysophosphatidic acid and pro-inflammatory and pro-calcifying signaling in the aortic valve [19].

OxLDL retained in valvular tissue can interact with scavenger receptors expressed on VICs and macrophages, promoting ROS production, inflammatory activation, and foam cell formation—processes that amplify local inflammation [65]. Furthermore, OxLDL and lysophosphatidylcholine present in modified apoB particles can provide substrates for ATX and enhance the local generation of LPC [59]. From a pathophysiological perspective, the interaction between Lp(a), oxLDL, and other lipoproteins with modified apoB increases the local load of OxLDL and bioactive lysolipids [18]. This convergence may amplify oxidative stress, inflammation, and osteogenic programming of valve cells, contributing to the fibrocalcifying progression of CAVD [19]. See Figure 3.

## 5. Clinical Evidence of the Association Between Lp(a) and Calcified Aortic Valve Disease

### 5.1. Genetic Association Studies

Variants of the *LPA* locus constitute one of the most consistent lines of evidence linking Lp(a) to CAVD [15]. The rs10455872 variant is associated with elevated plasma concentrations of Lp(a) and an approximately 2-fold increased risk of incident aortic stenosis, supporting a genetic relationship between Lp(a) and aortic valve disease [14]. This association was subsequently evaluated in a prospective Mendelian randomized study that included 17,553 participants and confirmed that genetically determined elevated Lp(a) levels are associated with an increased risk of incident aortic stenosis during a mean follow-up of 11.7 years [15]. Taken together, these findings reduce the likelihood that the association between Lp(a) and CAVD is solely due to residual confounding or reverse causality.

The variability in the number of KIV-2 repeats of the *LPA* gene is another relevant determinant of valvular risk [12,87,88]. Small apo(a) isoforms, associated with fewer KIV-2 repeats, are linked to higher Lp(a) concentrations and an increased risk of aortic stenosis, demonstrating a dose–response relationship between isoform size, plasma Lp(a) concentration, and valvular disease [87]. In patients with bicuspid aortic valves, a lower number of KIV-2 repeats has also been associated with more severe valvular calcification, suggesting that the genetic architecture of apo(a) may modulate susceptibility to fibrocalcifying progression in specific anatomical phenotypes [88]. Recent studies continue to support the association between elevated Lp(a) concentrations, *LPA* gene variants, and calcified aortic valve disease, although uncertainties remain regarding the magnitude of the effect in clinical subgroups and its relationship to individual hemodynamic progression [89].

### 5.2. Independence of Lp(a) from LDL-C and Persistence of Residual Risk

Lp(a) represents a source of cardiovascular and valvular risk that is partially independent of the conventional lipid profile, including LDL-C [9]. In patients with familial hypercholesterolemia, Lp(a) levels do not show a significant correlation with either LDL-C or HDL-C, supporting the hypothesis that these two lipoproteins are determined by partially distinct biological mechanisms [90]. This independence is also observed in genetic studies, in which variants of the *LPA* locus are associated with an increased risk of aortic calcification and stenosis, independent of LDL-C levels [14]. In this context, Lp(a) may contribute to residual risk through its own mechanisms, including OxPL transport, valvular inflammation, and procalcifying signaling [18].

LDL-C reduction with statins does not completely eliminate the risk associated with elevated Lp(a). A meta-analysis of individual data from six statin-controlled trials showed that Lp(a) concentrations >50 mg/dL were associated with increased cardiovascular risk, even among participants who achieved LDL-C < 70 mg/dL, supporting its contribution to residual risk (HR 1.38) [9]. Furthermore, Lp(a) concentrations exhibit high intraindividual stability and limited variability in serial measurements, reinforcing its usefulness as a predominantly genetic biomarker that can be assessed with a single determination in most patients [91,92]. This stability distinguishes it from other lipids that are more sensitive to metabolic and environmental changes and supports its inclusion in the risk stratification of patients susceptible to CAVD [7,9].

### 5.3. Computed Tomography and Echocardiography Evidence of the Association Between Lp(a) and CAVD

Computed tomography, using the Agatston score, has confirmed that elevated Lp(a) levels are independently associated with baseline valvular calcification [5]. In the Rotterdam Study, elevated Lp(a) concentrations were associated with both baseline and incident valvular calcification, after adjusting for conventional cardiovascular risk factors [17]. Similarly, the Copenhagen General Population Study showed a strong association between increased aortic valve calcification and LPA locus variants [12]. In MESA, elevated Lp(a) concentrations were associated with a higher incidence and annual progression of aortic valve calcification assessed by computed tomography, even after adjusting for conventional cardiovascular risk factors [93]. These findings suggest a predominant role of Lp(a) in the initial stages of calcification, before the disease becomes dominated by the established mineral burden [18].

Additionally, echocardiographic evidence reveals that Lp(a) is associated with faster hemodynamic progression of aortic stenosis [10,30]. In a meta-analysis of individual data that included 710 patients from five longitudinal cohorts, participants with higher Lp(a) concentrations had a greater annual increase in peak transvalvular velocity and mean gradient compared with those with lower levels [10]. Consistent results were observed in previous prospective studies, in which elevated Lp(a) was associated with greater echocardiographic progression, need for aortic valve replacement, and cardiovascular death [18,30]. Taken together, the findings from tomography and echocardiography suggest that Lp(a) is involved both in the initiation of valvular calcification and in the more unfavorable hemodynamic evolution of established disease [18].

### 5.4. Cardiac Magnetic Resonance Imaging, Lp(a) and CAVD

Cardiac magnetic resonance (CMR) imaging is a fundamental tool for characterizing myocardial architecture, allowing differentiation between focal fibrosis using late gadolinium enhancement (LGE) and diffuse interstitial fibrosis through T1 mapping and extracellular volume fraction [94]. In the context of aortic stenosis, replacement fibrosis identified by LGE is a marker of pathological ventricular remodeling and is associated with an unfavorable clinical prognosis [95]. Meanwhile, alterations in native T1 and extracellular volume can identify interstitial expansion at earlier stages, before focal fibrosis detectable by LGE is established [94]. Thus, CMR offers a comprehensive assessment of myocardial impact from pressure overload, accurately complementing the structural information and hemodynamic assessment obtained through computed tomography and echocardiography [96].

However, the association between Lp(a) and myocardial fibrosis varies significantly depending on the imaging technique used [97]. In this regard, smaller studies have not identified a clear relationship between Lp(a) and focal fibrosis due to LGE, possibly due to sensitivity limitations in early or diffuse phases [95]. However, an analysis of 2040 participants from the MESA study demonstrated an independent association between elevated Lp(a) levels and greater subclinical interstitial fibrosis as assessed by T1 mapping [97,98]. These findings suggest that Lp(a) is involved in diffuse fibrotic remodeling independently of overt valvular disease, reinforcing its role as a causal entity with its own pathophysiological mechanisms and underscoring the importance of T1 mapping for the early detection of these changes [97,99].

### 5.5. Molecular PET/CT to Evaluate Inflammation and Valvular Calcifying Activity (18F-FDG, NaF)

Positron Emission Tomography combined with Computed Tomography (PET/CT) allows evaluation of valvular biological activity before calcification is fully evident on conventional tomography [100]. 18F-fluorodeoxyglucose (18F-FDG) reflects an increase in glycolytic metabolism associated with inflammation, particularly in activated macrophages, while 18F-sodium fluoride (18F-NaF) binds to forming hydroxyapatite surfaces and allows for the identification of active microcalcification [101]. In calcified aortic stenosis, valvular uptake of 18F-NaF identifies active calcification and predicts subsequent disease progression, thereby serving as an in vivo biomarker of early mineralization [100]. This characteristic is particularly relevant in patients with elevated Lp(a), since their OxPL can promote calcifying activity before extensive macroscopic burden is present [18].

Clinical evidence directly links Lp(a), OxPL, and valvular calcifying activity measured by PET/CT [102]. In two prospective cohorts of patients with aortic stenosis, elevated Lp(a) and OxPL-apoB concentrations were associated with increased valvular uptake of 18F-NaF, accelerated progression of valvular calcium on CT, and greater hemodynamic deterioration during follow-up [18,103]. Although the combination of 18F-FDG and 18F-NaF can provide complementary information on inflammation and mineralization, the uptake of both tracers does not always coincide spatially, suggesting that inflammation and calcification may predominate in different phases or regions of the disease [104]. Therefore, PET/CT should currently be considered a research and biological stratification tool, rather than an established method for deciding on Lp(a) targeted interventions in clinical practice [18].

## 6. Therapeutic Implications

The recognition of Lp(a) as a genetic and potentially causal factor in calcified aortic valve disease has strengthened interest in targeted therapies in the early stages of the disease, before irreversible valvular obstruction [15]. In this context, the association between Lp(a), OxPL, and accelerated progression of aortic stenosis provides a mechanistic basis for considering Lp(a) reduction as a potential therapeutic strategy, beyond its value as a risk biomarker [30]. Molecular imaging studies have reinforced this hypothesis by demonstrating that Lp(a) and OxPL are related to valvular calcification activity, progression of calcium burden, and hemodynamic deterioration [18].

Emerging anti-Lp(a) therapies have demonstrated profound and sustained reductions in Lp(a), although their impact on valvular outcomes is not yet established. This is the case with pelacarsen [105] and olpasiran [106], as well as zerlasiran [107], muvalaplin [108], and lepodisiran [109]. If these reductions succeed in attenuating valvular inflammation, osteogenic differentiation of interstitial cells, and calcifying fibrous remodeling, they could delay progression to clinically significant aortic stenosis [30]. Therefore, early Lp(a) measurement could facilitate a primary prevention and precision medicine strategy in genetically susceptible individuals, especially before the development of advanced valvular calcification or the indication for valve replacement [18].

**Figure 4:**
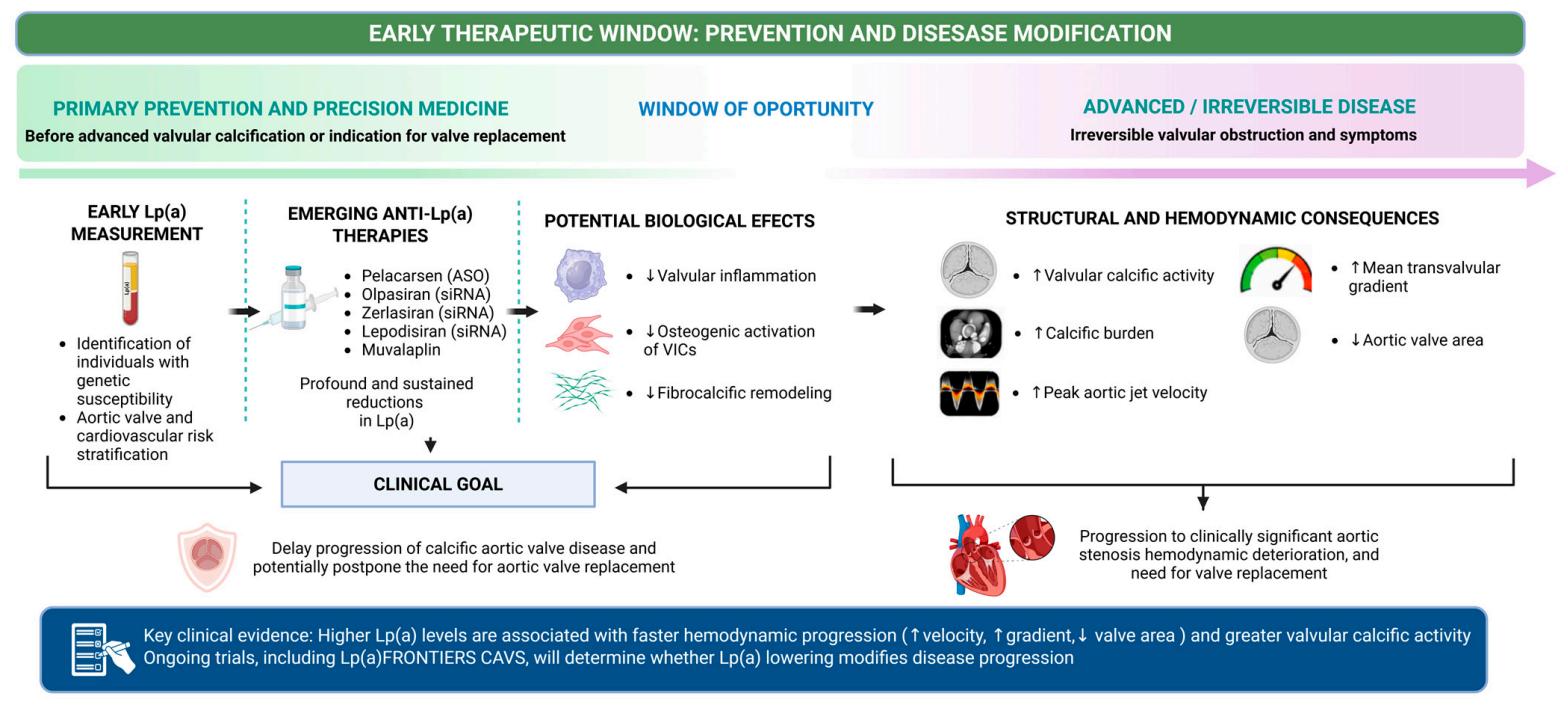
Early therapeutic window for Lp(a)-targeted prevention in calcific aortic valve disease. Early Lp(a) measurement may identify individuals at increased risk of calcific aortic valve disease before irreversible valvular obstruction develops. Emerging Lp(a)-lowering therapies may attenuate valvular inflammation, osteogenic activation of valvular interstitial cells, and fibrocalcific remodelling, potentially slowing structural and haemodynamic progression. Ongoing trials, including Lp(a)FRONTIERS CAVS, will determine whether Lp(a) reduction modifies disease progression and delays the need for aortic valve replacement. Abbreviations: ASO: antisense oligonucleotide; Lp(a): lipoprotein(a); siRNA: small interfering RNA; VICs: valvular interstitial cells. Created in BioRender. Osorio, E. (2026) https://BioRender.com/2y63zb7 (accessed on 25 June 2026); figure preparation was completed in June 2026.

Recent clinical evidence reinforces the therapeutic plausibility of this approach, as a meta-analysis of individual data in patients with aortic stenosis demonstrated that higher Lp(a) concentrations are associated with more rapid hemodynamic progression, as assessed by peak aortic jet velocity, mean transvalvular gradient, and aortic valve area [10]. Consequently, clinical trials currently targeting Lp(a) reduction, such as Lp(a)FRONTIERS CAVS with pelacarsen, represent a critical step in determining whether this strategy can modify the progression of calcified aortic valve disease, rather than merely reduce a lipid marker associated with risk [105]. Therefore, systematic Lp(a) measurement could be valuable not only for stratifying atherosclerotic cardiovascular risk but also for identifying individuals with greater susceptibility to calcified aortic valve disease, thus facilitating preventive interventions before the disease reaches advanced structural or clinically irreversible stages [7]. See Figure 4.

## 7. Translational Perspective and Future Directions

Integrating genetic information, circulating biomarkers, and molecular imaging can improve the identification of patients with biologically active calcified aortic valve disease [110]. Variants of the *LPA* locus, elevated Lp(a) concentrations, and oxidized phospholipid burden are associated with increased susceptibility to valvular calcification and progression of aortic stenosis, thus representing candidates for more accurate risk stratification models [14,15,30]. The incorporation of 18F-NaF PET/CT could provide information on valvular microcalcification activity, allowing for the differentiation of patients with anatomically stable disease from those with greater mineralization activity and a higher potential for progression [18,100].

The main translational challenge lies in determining whether the early identification of these phenotypes can lead to interventions capable of modifying the natural history of the disease. Therapies targeting Lp(a), along with strategies aimed at modulating OxPL, the autotaxin–lysophosphatidic acid axis, and osteogenic activation of VICs, represent potential approaches to limit fibrocalcifying progression [19,57,77]. However, a pharmacological reduction in Lp(a) has not yet been shown to prevent valvular calcification or delay the hemodynamic progression of aortic stenosis; therefore, clinical trials designed with specific valvular and imaging outcomes are needed.

## 8. Conclusions

Lipoprotein(a) is emerging as a relevant biological determinant in calcific aortic valve disease, beyond its role as a residual marker of cardiovascular risk. Genetic, clinical, molecular, and imaging evidence supports its association with increased valvular calcifying activity and more rapid hemodynamic progression of aortic stenosis. The retention of lipoprotein(a) and its oxidized phospholipids within valvular tissue may promote endothelial activation, recruitment of monocyte–macrophage lineage cells, and oxidative stress. Together, these processes favor both myofibroblastic and osteogenic differentiation of valvular interstitial cells, ultimately contributing to progressive valvular calcification. In particular, the autotaxin–lysophosphatidic acid axis, nuclear factor kappa B-dependent inflammatory pathways, and the release of procalcifying extracellular vesicles appear to contribute to fibrocalcific remodeling and hydroxyapatite formation. However, calcific aortic valve disease remains multifactorial, and a pharmacological reduction in lipoprotein(a) has not yet been shown to modify its clinical course. Therefore, trials with specific valvular outcomes are needed to determine whether early intervention targeting this lipoprotein can prevent or delay disease progression.

## Data Availability

No new data were created or analyzed in this study. Data sharing is not applicable to this article.

## Abbreviations

The following abbreviations are used in this manuscript:

ABCA1	ATP-binding cassette transporter A1
ABCG1	ATP-binding cassette transporter G1
ALP	alkaline phosphatase
apo(a)	apolipoprotein(a)
apoB	apolipoprotein B
apoB100	apolipoprotein B100
APOE	apolipoprotein E
ASCVD	atherosclerotic cardiovascular disease
ATX	autotaxin
BMP2	bone morphogenetic protein 2
BSCL2	BSCL2 lipid droplet biogenesis associated protein
CAC	coronary artery calcium
CAVD	calcified aortic valve disease
CCL2	C-C motif chemokine ligand 2
CMR	cardiac magnetic resonance
CX3CL1	C-X3-C motif chemokine ligand 1
ECM	extracellular matrix
EndMT	endothelial-to-mesenchymal transition
eNOS	endothelial nitric oxide synthase
EVs	extracellular vesicles
FDG	fluorodeoxyglucose
FOXS1	forkhead box S1
IL-1β	interleukin-1 beta
IL-6	interleukin-6
IL-6R	interleukin-6 receptor
IL-8	interleukin-8
JAK	Janus kinase
KIV	kringle IV
KIV-2	kringle IV type 2
LDL	low-density lipoprotein
LDL-R	LDL receptor
LGE	late gadolinium enhancement
LPAR1	lysophosphatidic acid receptor 1
LPAR3	lysophosphatidic acid receptor 3
LPC	lysophosphatidylcholine
Lp(a)	lipoprotein(a)
LXRα	liver X receptor alpha
MAPK	mitogen-activated protein kinase
MCP-1	monocyte chemoattractant protein-1
MESA	Multi-Ethnic Study of Atherosclerosis
NF-κB	nuclear factor kappa B
Nox2	NADPH oxidase 2
Nox4	NADPH oxidase 4
ox-LDL	oxidized low-density lipoprotein
OxPL	oxidized phospholipids
PET/CT	positron emission tomography/computed tomography
PI3K	phosphoinositide 3-kinase
PPARγ	peroxisome proliferator-activated receptor gamma
ROS	reactive oxygen species
RUNX2	runt-related transcription factor 2
RhoA	Ras homolog family member A
ROCK	Rho-associated coiled-coil-containing protein kinase
SR-A	scavenger receptor class A
SR-BI	scavenger receptor class B type I
STAT3	signal transducer and activator of transcription 3
TGF-β1	transforming growth factor beta 1
TNF-α	tumor necrosis factor alpha
VECs	valvular endothelial cells
VICs	valvular interstitial cells
